# Supplementary material for: End Sequence Analysis Toolkit (ESAT) expands the extractable information from single-cell RNA-seq data
Source: Genome Res. 2016 Oct;26(10):1397–410. doi: 10.1101/gr.207902.116 (PMC5052061; doi:10.1101/gr.207902.116)
Supplement: Supplemental Material [file supp_gr.207902.116_Supp_Materials.tar.gz › ESAT-esat_v0.1/doc/html/classbroad_1_1pda_1_1geneexpression_1_1agilent_1_1_agilent_probe.html]

ESAT: broad.pda.geneexpression.agilent.AgilentProbe Class Reference


ESAT


- Main Page
- Related Pages
- Packages
- Classes
- Files

- Class List
- Class Index
- Class Hierarchy
- Class Members

All Classes Namespaces Files Functions Variables Enumerator Pages

- broad
- pda
- geneexpression
- agilent
- AgilentProbe

Public Member Functions |
List of all members

broad.pda.geneexpression.agilent.AgilentProbe Class Reference

|  |  |
| --- | --- |
| Public Member Functions | |
|  | AgilentProbe (String line, String headerLine) |
|  | |
| String | getGeneName () |
|  | |
| String | getProbename () |
|  | |
| double | getProcessedSignal () |
|  | |
| int | getColumnNum () |
|  | |
| String | getGeneDescription () |
|  | |
| int | getRownum () |
|  | |
| boolean | isControlProbe () |
|  | |
| boolean | isProbeFound () |
|  | |
| boolean | isProbeNonUniform () |
|  | |
| boolean | isProbePopulationOutlier () |
|  | |
| boolean | isProbeWellAboveBackground () |
|  | |
| boolean | isSaturated () |
|  | |
| double | getNormalizedSignal (double normFactor, double spikeInLowerLimit, double saturationLevel) |
|  | |
| double | getNormalizedSignal (double spikeInLowerLimit, double saturationLevel) |
|  | |
| boolean | isFlagged (double spikeInLowerLimit) |
|  | |
| double | getLogProcessedSignal () |
|  | |

## Constructor & Destructor Documentation

|  |  |  |  |
| --- | --- | --- | --- |
| broad.pda.geneexpression.agilent.AgilentProbe.AgilentProbe | ( | String | *line*, |
|  |  | String | *headerLine* |
|  | ) |  |  |

## Member Function Documentation

|  |  |  |  |  |
| --- | --- | --- | --- | --- |
| int broad.pda.geneexpression.agilent.AgilentProbe.getColumnNum | ( |  | ) |  |

|  |  |  |  |  |
| --- | --- | --- | --- | --- |
| String broad.pda.geneexpression.agilent.AgilentProbe.getGeneDescription | ( |  | ) |  |

|  |  |  |  |  |
| --- | --- | --- | --- | --- |
| String broad.pda.geneexpression.agilent.AgilentProbe.getGeneName | ( |  | ) |  |

|  |  |  |  |  |
| --- | --- | --- | --- | --- |
| double broad.pda.geneexpression.agilent.AgilentProbe.getLogProcessedSignal | ( |  | ) |  |

|  |  |  |  |
| --- | --- | --- | --- |
| double broad.pda.geneexpression.agilent.AgilentProbe.getNormalizedSignal | ( | double | *normFactor*, |
|  |  | double | *spikeInLowerLimit*, |
|  |  | double | *saturationLevel* |
|  | ) |  |  |

|  |  |  |  |
| --- | --- | --- | --- |
| double broad.pda.geneexpression.agilent.AgilentProbe.getNormalizedSignal | ( | double | *spikeInLowerLimit*, |
|  |  | double | *saturationLevel* |
|  | ) |  |  |

|  |  |  |  |  |
| --- | --- | --- | --- | --- |
| String broad.pda.geneexpression.agilent.AgilentProbe.getProbename | ( |  | ) |  |

|  |  |  |  |  |
| --- | --- | --- | --- | --- |
| double broad.pda.geneexpression.agilent.AgilentProbe.getProcessedSignal | ( |  | ) |  |

|  |  |  |  |  |
| --- | --- | --- | --- | --- |
| int broad.pda.geneexpression.agilent.AgilentProbe.getRownum | ( |  | ) |  |

|  |  |  |  |  |
| --- | --- | --- | --- | --- |
| boolean broad.pda.geneexpression.agilent.AgilentProbe.isControlProbe | ( |  | ) |  |

|  |  |  |  |  |  |
| --- | --- | --- | --- | --- | --- |
| boolean broad.pda.geneexpression.agilent.AgilentProbe.isFlagged | ( | double | *spikeInLowerLimit* | ) |  |

Here is the call graph for this function:

|  |  |  |  |  |
| --- | --- | --- | --- | --- |
| boolean broad.pda.geneexpression.agilent.AgilentProbe.isProbeFound | ( |  | ) |  |

|  |  |  |  |  |
| --- | --- | --- | --- | --- |
| boolean broad.pda.geneexpression.agilent.AgilentProbe.isProbeNonUniform | ( |  | ) |  |

Here is the caller graph for this function:

|  |  |  |  |  |
| --- | --- | --- | --- | --- |
| boolean broad.pda.geneexpression.agilent.AgilentProbe.isProbePopulationOutlier | ( |  | ) |  |

|  |  |  |  |  |
| --- | --- | --- | --- | --- |
| boolean broad.pda.geneexpression.agilent.AgilentProbe.isProbeWellAboveBackground | ( |  | ) |  |

Here is the caller graph for this function:

|  |  |  |  |  |
| --- | --- | --- | --- | --- |
| boolean broad.pda.geneexpression.agilent.AgilentProbe.isSaturated | ( |  | ) |  |

---

The documentation for this class was generated from the following file:

- java/broad/pda/geneexpression/agilent/AgilentProbe.java


---

Generated on Fri Aug 15 2014 15:11:00 for ESAT by  

 1.8.7
